# Macrophage C/EBPδ Drives Gemcitabine, but Not 5-FU or Paclitaxel, Resistance of Pancreatic Cancer Cells in a Deoxycytidine-Dependent Manner

**DOI:** 10.3390/biomedicines10020219

**Published:** 2022-01-20

**Authors:** C. Arnold Spek, Hella L. Aberson, JanWillem Duitman

**Affiliations:** 1 Center for Experimental and Molecular Medicine, Amsterdam UMC, University of Amsterdam, 1105 AZ Amsterdam, The Netherlands; h.l.aberson@amsterdamumc.nl; 2 Department of Respiratory Medicine, Amsterdam UMC, University of Amsterdam, 1105 AZ Amsterdam, The Netherlands; j.w.duitman@amsterdamumc.nl

**Keywords:** CCAAT/enhancer-binding protein delta, CEBPD, gemcitabine, drug resistance, pancreatic cancer, PDAC

## Abstract

Treatment of pancreatic ductal adenocarcinoma (PDAC), a dismal disease with poor survival rates, is hampered by the high prevalence of chemotherapy resistance. Resistance is accompanied by macrophage infiltration into the tumor microenvironment, and infiltrated macrophages are key players in chemotherapy resistance. In the current manuscript, we identify CCAAT/enhancer-binding protein delta (C/EBPδ) as an important transcription factor driving macrophage-dependent gemcitabine resistance. We show that conditioned medium obtained from wild type macrophages largely diminishes gemcitabine-induced cytotoxicity of PDAC cells, whereas conditioned medium obtained from C/EBPδ-deficient macrophages only minimally affects gemcitabine-induced PDAC cell death. Subsequent analysis of RNA-Seq data identified the pyrimidine metabolism pathway amongst the most significant pathways down-regulated in C/EBPδ-deficient macrophages and size filtration experiments indeed showed that the low molecular weight and free metabolite fraction most effectively induced gemcitabine resistance. In line with a role for pyrimidines, we next show that supplementing macrophage conditioned medium with deoxycytidine overruled the effect of macrophage conditioned media on gemcitabine resistance. Consistently, macrophage C/EBPδ-dependent resistance is specific for gemcitabine and does not affect paclitaxel or 5-FU-induced cytotoxicity. Overall, we thus show that C/EBPδ-dependent deoxycytidine biosynthesis in macrophages induces gemcitabine resistance of pancreatic cancer cells.

## 1. Introduction

Pancreatic adenocarcinoma (PDAC) is a devastating disease with one of the worst survival rates of all human cancers [1]. Despite improvements in the treatment of cancer in general, hardly any progress has been made in PDAC treatment and the average survival rate remains around 6-8 months. Five years after diagnosis, more than 90% of PDAC patients have died, while 10-year mortality rates approach 99% [2,3]. The dismal prognosis of PDAC patients is largely due to the fact that most patients present with locally advanced or metastatic disease and are consequently ineligible for curative resection [4]. Chemotherapy-based therapies therefore remain standard of care [5], but chemotherapeutic treatments only minimally affect PDAC progression [6,7], therefore improving the efficacy of chemotherapy in PDAC is of utmost importance.

Macrophages are specialized phagocytic cells of the innate immune system critically involved in host defense and tissue homeostasis [8]. In cancer biology, macrophages are traditionally considered anti-tumorigenic, as seminal papers showed that macrophages may kill tumor cells by secreting cytotoxic molecules, such as TNF-α, IL-12, nitric oxide (NO) and reactive oxygen species (ROS) [9,10]. Once recruited to the tumor, however, macrophages are reprogrammed to prevent killing of cancer cells. Such reprogrammed M2 or tumor associated macrophages (TAMs) not only show limited cytotoxicity towards cancer cells but actually become pro-tumorigenic and potentiate tumor growth [11,12,13]. Indeed, TAMs induce epithelial-to-mesenchymal transition (EMT) [14,15,16] and limiting macrophage infiltration decreases the number of metastatic lesions [17]. In addition, TAM numbers are associated with therapy resistance in PDAC [18,19], and pharmacological depletion of TAMs enhanced the therapeutic response to gemcitabine in tumor-bearing KPC mice [20].

CCAAT/enhancer-binding protein delta (C/EBPδ), also known as nuclear factor interleukin (IL)-6β, is a member of the C/EBP family of transcription factors [21]. It modulates many biological processes involved in cancer biology and C/EBPδ acts as tumor suppressor in multiple tumor types [22,23,24,25]. In PDAC, C/EBPδ is down-regulated in cancer cells, and reduced C/EBPδ expression correlates with poor prognosis [26]. As opposed to low C/EBPδ expression in PDAC cells, most stromal cells show strong C/EBPδ expression [27]. In particular, the expression of C/EBPδ in macrophages seems interesting, as C/EBPδ is well-known to regulate macrophage-dependent chemokine and cytokine expression [28], whereas C/EBPδ also may affect macrophage polarization [29]. Considering the importance of cytokine production and macrophage polarization in drug resistance [30], we hypothesized that macrophage C/EBPδ would have an impact on drug resistance of PDAC cells. By using C/EBPδ-deficient macrophages, we show that macrophage C/EBPδ drives gemcitabine, but not 5-FU or paclitaxel, resistance in a deoxycytidine-dependent manner.

## 2. Materials and Methods

### 2.1. Cell Culture

PANC-1, MIA PaCa-2 and BxPc3 PDAC cells (ATCC, Manassas, VA, USA) were cultured in DMEM (Lonza, Basel, Switzerland), whereas wild type and C/EBPδ knock out RAW264.7 macrophages, generated and characterized as described previously [31], were cultured in IMDM medium (Gibco, Thermo Fischer Scientific, Waltham, MA, USA). All media were supplemented with 10% fetal calf serum (FCS), 2 mM L-glutamine, 100 units/mL penicillin, and 500 μg/mL streptomycin (all Lonza, Basel, Switzerland) according to routine cell culture procedures. Cells were maintained in a humidified incubator at 37 °C and 5% CO2. All PDAC cell lines were authenticated by STR profiling (Promega PowerPlex, Leiden, Netherlands), and tested for mycoplasma by PCR monthly.

### 2.2. Conditioned Medium Collection and PDAC Cytotoxicity Assays

Wild type and C/EBPδ knock out RAW264.7 macrophages were grown to 70% confluence in a T75 flask, after which the growth medium was refreshed. After 24 h, medium was collected, sterilized through a 0.2 µm filter and stored at −20 °C. PDAC cells, seeded in 100 μL complete medium in 96-well plates were, upon adherence of the cells, incubated in conditioned medium (diluted 1:1 in complete DMEM) supplemented with gemcitabine, 5-FU or paclitaxel. Based on IC50 analysis (Figures S1 and S3), PANC-1 cells were treated with 20 nM gemcitabine, 5 nM paclitaxel or 4 μM 5-FU, MIA PaCa-2 cells were treated with 15 nM gemcitabine, 20 nM paclitaxel or 2 μM 5-FU, whereas BxPc3 cells were treated with 10 nM gemcitabine, 4 nM paclitaxel or 4 μM 5-FU. After 96 h, drug-induced cytotoxicity was assessed essentially as described before [32]. In detail, PDAC cells were washed and incubated with crystal violet (3% formaldehyde, 0.5% crystal violet in H_2_O) at room temperature. After 20 min, the crystal violet solution was removed, cells were washed 3 times with tap water, and 75 µL/well DMSO was added to solubilize the formed crystals. After 20 min of incubation on a plate shaker at room temperature, the absorbance was measured at 600 nm on a Synergy HT plate reader (BioTek Instruments, Winooski, VT, USA).

### 2.3. Size Separation of Proteins in Conditioned Media

RAW264.7 conditioned media, collected as described above, were separated using 10 kD Molecular Weight Cut Off ultra-centrifugal filters (Amicon-Ultra, Millipore, Ireland). The concentrated >10 kD fraction was resuspended in IMDM to the original volume before storage at 4 °C, whereas the flow-through fraction (<10 kD) was stored at 4 °C until used in experiments.

### 2.4. RT-qPCR and RNA-Seq

RNA was extracted form TriReagent lysed cells according to routine procedures. Eluted RNA was analyzed spectrophotometrically using a NanoDrop 2000 (Thermo Fisher Scientific, Waltham, MA, USA). All samples were treated with RQ1 RNAse-Free DNAse (Promega Benelux BV) and reverse-transcribed into cDNA using M-MLV Reverse Transcriptase (Promega Benelux BV, Leiden, The Netherlands), random hexamers (Fisher scientific) and 10 mM dNTPs (Fermentas, Fisher scientific, Landsmeer, The Netherlands). The Sensi-FAST™ SYBR^®^ No-ROX Kit (GC biotech, Waddinxveen, The Netherlands) was used to perform real-time quantitative RT-PCR on a LightCycler^®^ 480 Instrument II (Roche Molecular Systems, Inc., Almere, The Netherlands). Expression levels were normalized to the expression of the reference genes TBP, B2M and UBC using the primers listed in Supplementary Table S1. RNA-Seq results and the corresponding experimental design were described previously [31] and represent transcriptional differences between wild type and C/EBPδ-deficient RAW264.7 cells. The complete sequence libraries are publicly available through the National Center for Biotechnology Information gene expression omnibus under the following accession number: GSE173552. Differential gene expression was analyzed using the R2 microarray analysis and visualization platform (R2: Genomics Analysis and Visualization Platform. Available online: http://r2.amc.nl; last accessed 12 January 2022) with a false discovery adjusted *p*-value less than 0.01. To analyze the function of the differentially expressed genes, Kyoto Encyclopedia of Genes and Genomes (KEGG) pathway enrichment analyses were conducted with a *p*-value cutoff off less than 0.005.

### 2.5. Statistical Analysis

All data are expressed as means ± SEM. Differences between multiple groups were analyzed by one-way ANOVA with Bonferroni correction for multiple testing, whereas t-tests were used for comparisons between 2 groups. Analyses were performed using GraphPad Prism version 8 (GraphPad Software, Inc., La Jolla, CA, USA). Statistically significant differences were considered with a *p*-value less than 0.05.

## 3. Results

### 3.1. Macrophage C/EBPδ Drives Gemcitabine Resistance of Pancreatic Cancer Cells

**Figure 1:**
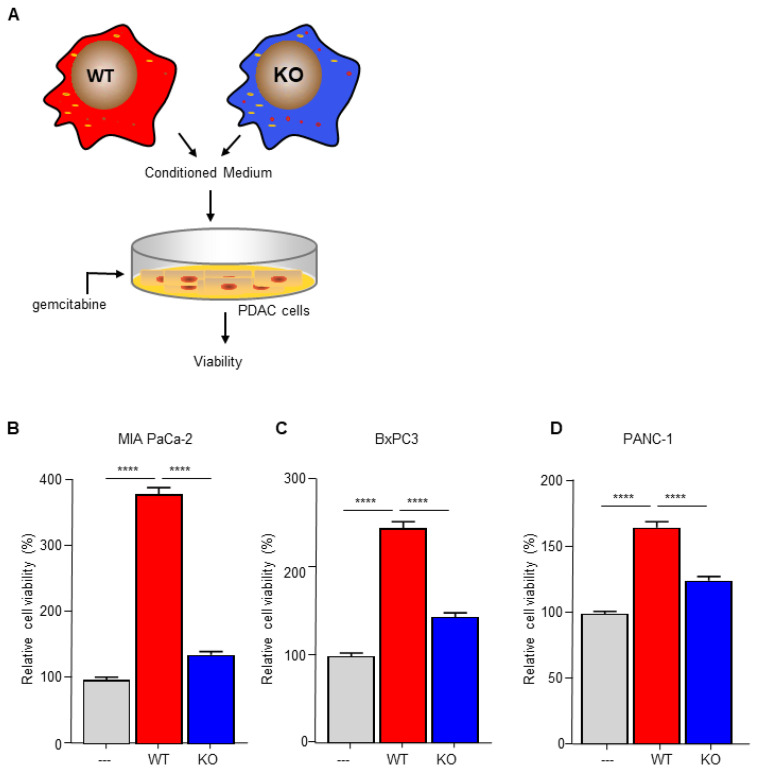
Macrophage C/EBPδ drives gemcitabine resistance of pancreatic cancer cells. (**A**) Schematic overview of the experimental set up. (**B–D**) Viability of Mia PaCa-2 (B), BxPc3 (**C**) and PANC-1 (**D**) pancreatic cancer cells in control medium (---), conditioned medium obtained from wild type macrophages (WT) and conditioned medium obtained from C/EBPδ-deficient macrophages (KO) in the presence of gemcitabine. Shown is the mean ± SEM of two to three independent experiments performed in sixplo. **** *p* < 0.0001.

To investigate the importance of C/EBPδ in macrophage-dependent resistance to gemcitabine, we employed wild type and C/EBPδ-deficient RAW264.7 macrophages. Conditioned medium of these cells was compared to control medium in gemcitabine-induced pancreatic cancer cell cytotoxicity assays (Figure 1A). As shown in Figure 1B–D, conditioned medium obtained from wild type macrophages induced gemcitabine resistance in MIA PaCa-2 (Figure 1B), BxPc3 (Figure 1C) and PANC-1 (Figure 1D) cells. Of note, conditioned medium obtained from C/EBPδ-deficient macrophages did not affect gemcitabine-induced cytotoxicity of any of the pancreatic cancer cells tested. To exclude a direct effect of conditioned medium on cell viability, MIA PaCa-2 cells were incubated in conditioned medium in the absence of gemcitabine, showing that conditioned medium indeed did not affect cell viability by itself (Figure S2).

### 3.2. C/EBPδ Deficiency Inhibits Pyrimidine Syntheses in Macrophages

**Figure 2:**
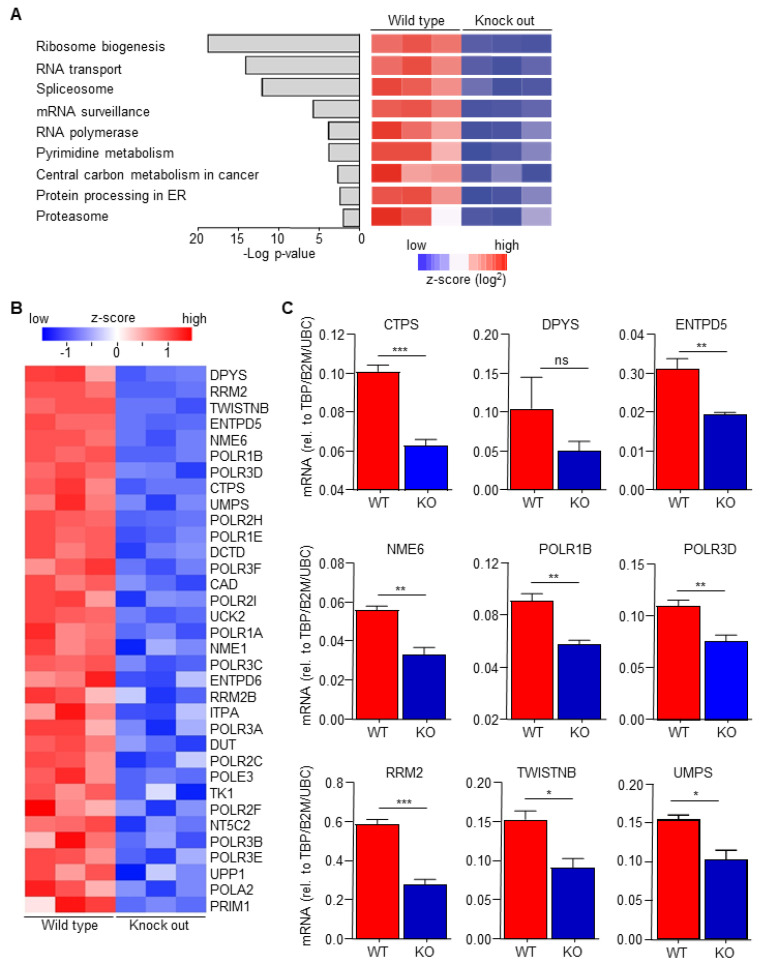
C/EBPδ deficiency inhibits pyrimidine syntheses. (**A**) The *p*-values and names of the most over-represented KEGG pathways, calculated on the basis of all the differentially expressed genes between wild type and C/EBPδ-deficient macrophages. (**B**) Heatmap of all the differentially expressed genes from the pyrimidine metabolism pathway. (**C**) RT-PCR validation of the top differentially expressed genes of the pyrimidine metabolism pathway. * *p* < 0.05; ** *p* < 0.01; *** *p* < 0.005; ns: not significant.

Macrophage-secreted chemokines and growth factors are suggested to induce drug resistance [18], suggesting that macrophage C/EBPδ potentiates gemcitabine resistance by enhancing chemokine/cytokine secretion. Analysis of recently obtained RNA-Seq data to determine transcriptional changes induced by C/EBPδ deletion in macrophages (GSE173552 [31]) revealed, however, no apparent changes in the expression of chemokines/cytokines. KEGG pathway enrichment analyses of differentially expressed genes between wild type and C/EBPδ-deficient macrophages, in fact, identified the pyrimidine metabolism pathway amongst the most significant pathways down-regulated by C/EBPδ deficiency (Figure 2A). Detailed analysis of the pyrimidine metabolism pathway showed that 34 genes were significantly decreased in C/EBPδ-deficient macrophages (Figure 2B) of which the 9 most down-regulated genes were confirmed by RT-PCR analysis (Figure 2C).

### 3.3. Macrophage C/EBPδ Drives Gemcitabine Resistance in a Deoxycytidine-Dependent Manner

**Figure 3:**
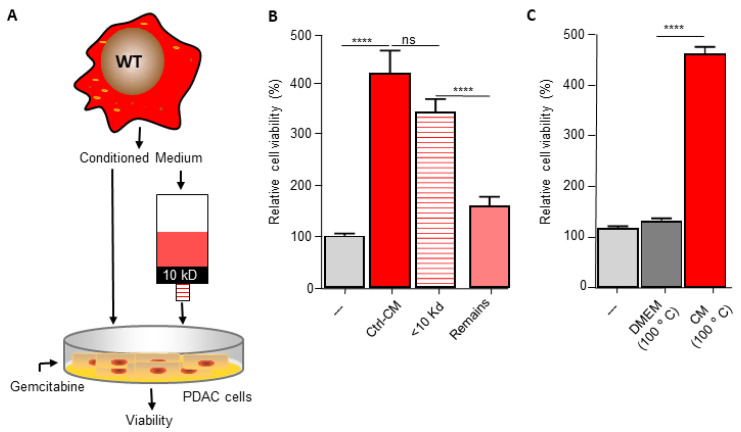

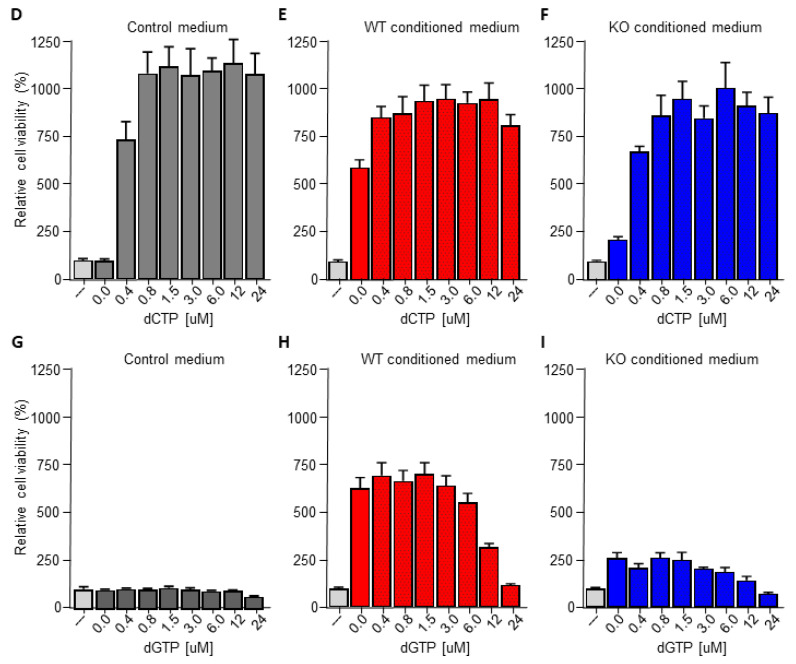
Macrophage C/EBPδ drives gemcitabine resistance in a deoxycytidine-dependent manner. (**A**) Schematic overview of the experimental set up. (**B**) Viability of Mia PaCa-2 pancreatic cancer cells in control medium (---), unfractionated conditioned medium obtained from wild type macrophages (Ctrl-CM), in the flow through fraction containing free metabolites (<10 kD) and in the fraction that retained on the filter (remains) in the presence of gemcitabine. **** *p* < 0.0001; ns: not significant. (**C**) Viability of Mia PaCa-2 pancreatic cancer cells in control medium (---), boiled DMEM (DMEM 100 °C) or boiled conditioned medium obtained from wild type macrophages (CM 100 °C) in the presence of gemcitabine. **** *p* < 0.0001. (**D**–**F**) Viability of Mia PaCa-2 pancreatic cancer cells in control medium (**D**) or conditioned medium obtained from wild type (**E**) or C/EBPδ-deficient macrophages (**F**) in the presence of gemcitabine and the indicated concentration of deoxycytidine (dCTP). (**G**–**I**) Viability of Mia PaCa-2 pancreatic cancer cells in control medium (**G**) or conditioned medium obtained from wild type (**H**) or C/EBPδ-deficient macrophages (**I**) in the presence of gemcitabine and the indicated concentration of deoxycytidine (dCTP). Shown is the mean ± SEM of experiments performed in sixplo. Note that the light grey bars in panels (**D**–**I**) (---) represent cell viability in control conditions (i.e., control medium supplemented with gemcitabine).

The pyrimidine metabolism pathway has previously been shown to affect gemcitabine resistance [33,34]. Indeed, deoxycytidine directly competes with gemcitabine thereby inhibiting its uptake and activation, leading to diminished intracellular gemcitabine activity and reduced gemcitabine-induced cytotoxicity [33]. It is thus tempting to speculate that macrophage C/EBPδ drives gemcitabine resistance in a deoxycytidine-dependent manner. To prove or refute this hypothesis, we first subjected conditioned medium obtained from wild type macrophages to a size filtration spin column with a 10 kD cutoff to separate proteins from free metabolites (Figure 3A). As shown in Figure 3B, the flow through the fraction containing free metabolites was almost as effective in inducing gemcitabine resistance as control, non-filtrated, conditioned medium. The fraction that retained on the filter (containing proteins but also residual metabolites) was clearly less effective in inducing gemcitabine resistance of pancreatic cancer cells. In line with these findings that suggest that the resistance-inducing factor is not a protein, heat denaturation of the conditioned medium (15 min 100 °C) prior to transfer to pancreatic cancer cells did not diminish gemcitabine resistance (Figure 3C). Based on a model in which C/EBPδ-dependent deoxycytidine biosynthesis in macrophages induces gemcitabine resistance of pancreatic cancer cells, exogenous administration of deoxycytidine should overrule the effect of macrophage C/EBPδ. As shown in Figure 3D–F, deoxycytidine co-treatment indeed limits gemcitabine-induced cytotoxicity to a similar extent in control, wild type and C/EBPδ-deficient conditioned medium. Of note, supplementing control or C/EBPδ-deficient conditioned medium with low dose deoxycytidine (0.4 uM) already results in similar gemcitabine resistance as induced by wild type conditioned medium (compare the 0.4 uM bars of panels D and F with the 0 uM bar of panel E). Supplementing control or conditioned medium with the pyrimidine deoxyguanosine did not induce gemcitabine resistance (Figure 3G–I), in line with the notion that deoxyguanosine does not compete with gemcitabine uptake and activation.

### 3.4. Macrophage C/EBPδ-Dependent Resistance of Pancreatic Cancer Cells Is Specific for Deoxycytidine Analogs

**Figure 4:**
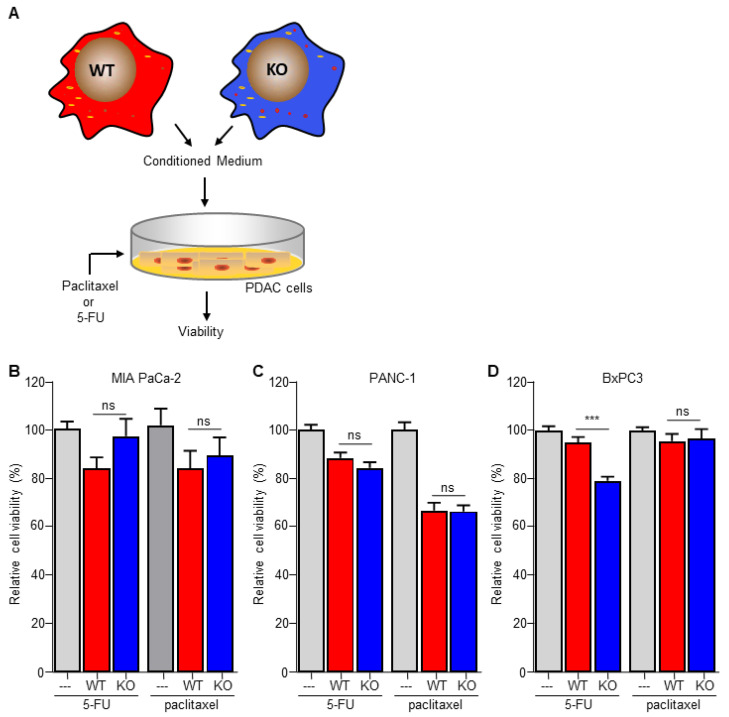
Macrophage C/EBPδ selectively induces resistance to gemcitabine. (**A**) Schematic overview of the experimental set up. (**B**–**D**) Viability of Mia PaCa-2 (**B**), BxPc3 (**C**) and PANC-1 (**D**) pancreatic cancer cells in control medium (---), conditioned medium obtained from wild type macrophages (WT) and conditioned medium obtained from C/EBPδ-deficient macrophages (KO) in the presence of 5-FU or paclitaxel. Shown is the mean ± SEM of an experiment performed in sixplo. *** *p* < 0.0001; ns: not significant.

The data above suggest that macrophage C/EBPδ induces gemcitabine resistance by potentiating deoxycytidine biosynthesis and secretion. For this hypothesis to be true, macrophage C/EBPδ-induced resistance should be limited to gemcitabine. Consequently, we next assessed the effect of macrophage C/EBPδ on paclitaxel-, a cytotoxic agent routinely used in pancreatic cancer treatment that acts independent of DNA replication, and 5-FU-, a pyrimidine nucleoside-based cytotoxic agent with different transport and activation properties as deoxycytidine [33], induced cell death (Figure 4A). In line with our hypothesis, conditioned medium obtained from both wild type and C/EBPδ-deficient macrophages did not induce resistance to 5-FU or paclitaxel in MIA PaCa-2 (Figure 4B), PANC-1 (Figure 4C) or BxPc3 (Figure 4D) cells. Of note, conditioned medium did slightly enhance 5-FU- and paclitaxel-induced cytotoxicity in PANC1 and BxPc3 cells. Except for 5-FU-treated BxPc3 cells, this effect was similar for conditioned medium obtained from wild type and C/EBPδ-deficient macrophages.

## 4. Discussion

In the current study, we aimed to elucidate the importance of macrophage C/EBPδ in drug resistance of pancreatic cancer cells. We confirm that macrophage-derived conditioned medium induces gemcitabine resistance of pancreatic cancer cells and show that conditioned medium obtained from C/EBPδ-deficient macrophages no longer induces gemcitabine resistance. The fact that resistance, which occurs in most PDAC patients within weeks of treatment initiation, profoundly limits the efficacy of gemcitabine treatment [35], underscores the potential importance of our findings and suggests that C/EBPδ is a key factor underlying the poor prognosis of PDAC patients.

Single agent gemcitabine chemotherapy has been the backbone of PDAC treatment for many years [36,37,38,39], but combination therapy with folinic acid, fluorouracil, irinotecan and oxaliplatin (FOLFIRINOX) recently replaced gemcitabine as first-line treatment modality [40]. The increased clinical benefit of FOLFIRINOX comes, however, at the expense of increased toxicity [41]. In view of the substantial toxicity of FOLFIRINOX, understanding the underlying mechanisms by which PDAC patients become resistant to gemcitabine is not just of scientific importance. The identification of C/EBPδ as a driver of macrophage-dependent gemcitabine resistance suggests that patients with low C/EBPδ expression in their macrophages may be particularly eligible for gemcitabine treatment. On the contrary, patients with high macrophage C/EBPδ levels should preferably not be treated with gemcitabine, as they are prone to be resistant and experience limited benefit of treatment. Future studies should prove or refute the validity of this tantalizing hypothesis.

Macrophage numbers in or around tumors are typically associated with poor prognosis and reduced overall survival [11,12,13]. In line with this, pharmacological macrophage depletion was recently shown to enhance the therapeutic response to gemcitabine in a preclinical murine PDAC model [20]. These data not only confirm the general concept that macrophages, despite their cytotoxic nature, exert a pro-tumorigenic role [42] but also suggest that macrophage depletion could be an attractive option to increase the clinical efficacy of gemcitabine. Of note, however, is that specific macrophage subsets may possess tumor-suppressive functions based upon which it has been suggested that macrophage reprogramming rather than depletion may be the most promising strategy to pursue [20,43]. We identify C/EBPδ as an attractive candidate to reprogram macrophages in the setting of gemcitabine therapy in PDAC patients. Our data suggest that combination therapy of gemcitabine and a C/EBPδ inhibitor will enhance gemcitabine efficacy and will improve treatment response in PDAC patients. In particular, patients with high C/EBPδ positive macrophage numbers are likely to benefit from combination therapy, but this hypothesis needs to be addressed in preclinical PDAC models before one could pursue to clinical studies. Although no C/EBPδ inhibitor has yet been clinically approved, a recent study showed that two histone deacetylase (HDAC) inhibitors, suberoylanilide hydroxamic acid (SAHA) and trichostatin A (TSA), abolished endogenous C/EBPδ mRNA expression levels in THP-1 macrophages [44]. Whether these HDAC inhibitors do reduce C/EBPδ levels in tumor associated macrophages and subsequently potentiate gemcitabine efficacy in PDAC remains to be established in future experiments.

C/EBPδ plays a pleiotropic role in cancer biology and may either potentiate or inhibit cancer progression in a context-dependent manner. In PDAC, tumor cell C/EBPδ expression is lost and re-expression of C/EBPδ in PDAC cell lines slowed down proliferation and decreased the clonogenic capacity [26]. In contrast, however, primary tumor growth was not affected in C/EBPδ-deficient mice but metastases were observed in numerous organs of tumor cell grafted wild type mice but not in C/EBPδ-deficient mice [27]. Here, we extend the notion that C/EBPδ seems to exert opposing effects in tumor and non-tumor cells by showing that macrophage C/EBPδ limits the cytotoxicity of gemcitabine towards pancreatic cancer cells. Of note, a previous study suggested that macrophage C/EBPδ induced chemoresistance of breast cancer cells to both cisplatin and 5-FU [45]. The latter is especially interesting as we here show that wild type and C/EBPδ-deficient macrophages both do not induce resistance of pancreatic cancer cells towards 5-FU, further underscoring the context-dependent role of C/EBPδ in tumor biology.

Gemcitabine resistance of pancreatic cancer cells is multifactorial [46] and may result from pancreatic cancer cell autonomous processes, for instance mitochondria-mediated apoptosis [47,48] or nucleoside transporter down-regulation [49], and from interactions between pancreatic cancer cells and stromal cells. Tumor associated macrophages may induce gemcitabine resistance of pancreatic cancer cells by several mechanisms (excellently reviewed by Yang and colleagues [50]). Most importantly for the current study, macrophages release a spectrum of pyrimidine species of which deoxycytidine molecularly competes with gemcitabine uptake and metabolism thereby hindering its efficacy as a chemotherapy in PDAC [34]. We here confirm the importance of macrophage-derived deoxycytidine in gemcitabine resistance and identify C/EBPδ as a key transcription factor driving deoxycytidine biosynthesis in macrophages. In line with its specific effect on deoxycytidine biosynthesis, macrophage C/EBPδ did not impact resistance to either 5-FU or paclitaxel. Indeed, conditioned medium obtained from both wild type and C/EBPδ-deficient macrophages did not induce resistance to 5-FU and paclitaxel, but actually slightly enhanced 5-FU- and paclitaxel-induced cytotoxicity in PANC-1 and BxPc3 cells. At this moment, we do not have an explanation for the small additive effect of macrophage conditioned medium on 5-FU- and paclitaxel-induced cytotoxicity, but most importantly our data highlight that C/EBPδ specifically acts upon gemcitabine resistance of pancreatic cancer cells and that C/EBPδ is not a generic player in PDAC chemoresistance. Interestingly, however, pancreatic cancer cells are more resistant to gemcitabine than other chemotherapeutic drugs [46], making C/EBPδ an attractive transcription factor in the setting of chemoresistance in PDAC.

## 5. Conclusions

In conclusion, we here show that macrophage C/EBPδ drives gemcitabine resistance of pancreatic cancer cells in a deoxycytidine-dependent manner. Our data contribute to a better understanding of gemcitabine resistance in PDAC which may ultimately aid in improved prognosis of this dismal disorder.

## Data Availability

The data presented in this study are available from the corresponding author upon reasonable request.

## Supplementary Materials

The following supporting information can be downloaded at: www.mdpi.com/article/10.3390/biomedicines10020219/s1, Figure S1: Dose–response curves of gemcitabine treatment, Figure S2: Conditioned medium does not affect cell viability, Figure S3: Dose–response curves of paclitaxel and 5-FU treatment, Table S1: Primers for LC validation.
