# Supplementary material for: Macrophage C/EBPδ Drives Gemcitabine, but Not 5-FU or Paclitaxel, Resistance of Pancreatic Cancer Cells in a Deoxycytidine-Dependent Manner
Source: Biomedicines. 2022 Jan 20;10(2):219. doi: 10.3390/biomedicines10020219 (PMC8869168; doi:10.3390/biomedicines10020219)
Supplement: Supplementary file 1 [file biomedicines-10-00219-s001.zip › biomedicines-1540438-supplementary proof done.pdf]

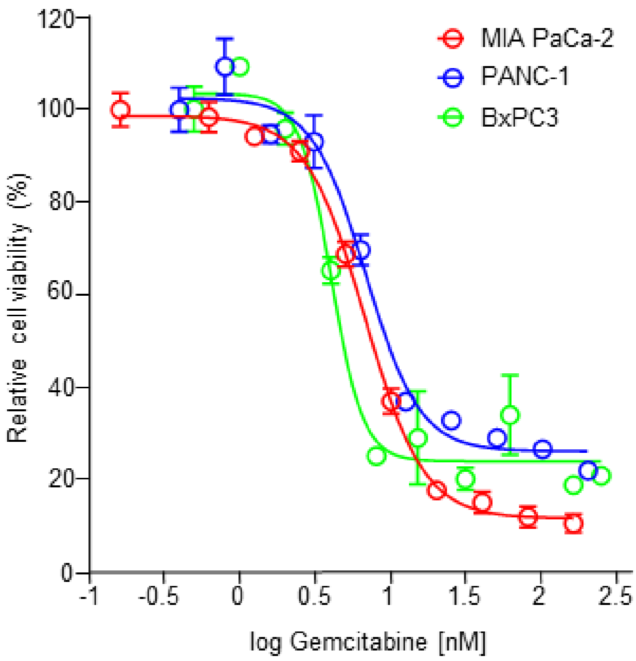

Supplemental Figure S1: Dose-response curves of gemcitabine treatment. Cell Mia Paca-2, BxPc3 and PANC-1 cells at 96 hours of drug exposure. Shown is the mean±SEM of a representative experiment performed in sixplo.

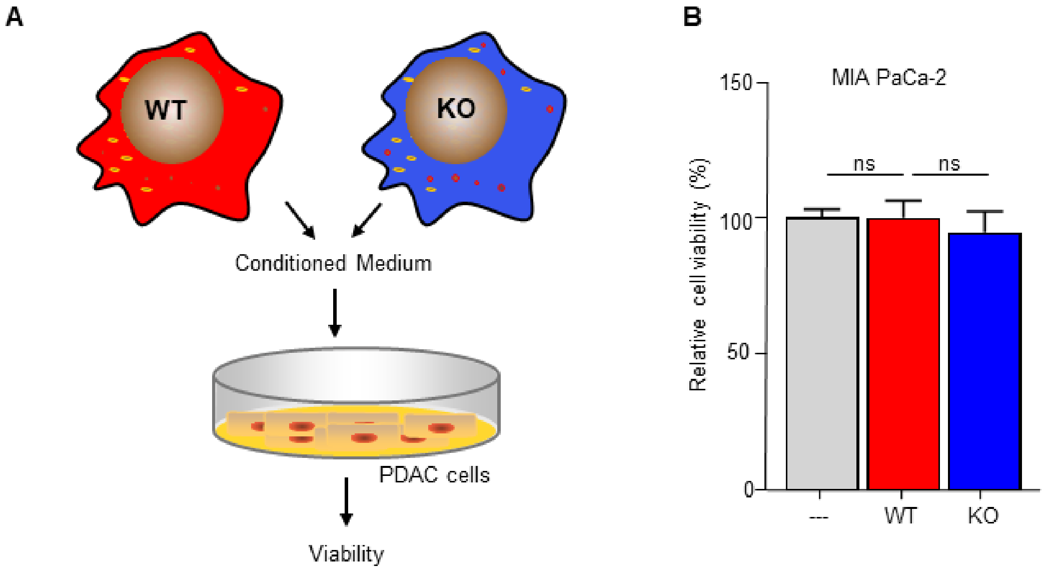

Supplemental Figure S2: Conditioned medium does not affect cell viability. (A) Schematic overview of the experimental set up. (B) Viability of Mia PaCa-2 pancreatic cancer cells in control medium (---), conditioned medium obtained from wild type (WT) macrophages and conditioned medium obtained from C/EBPδ deficient (KO) macrophages. Shown is the mean±SEM of an experiment performed in sixplo.ns: not significant.

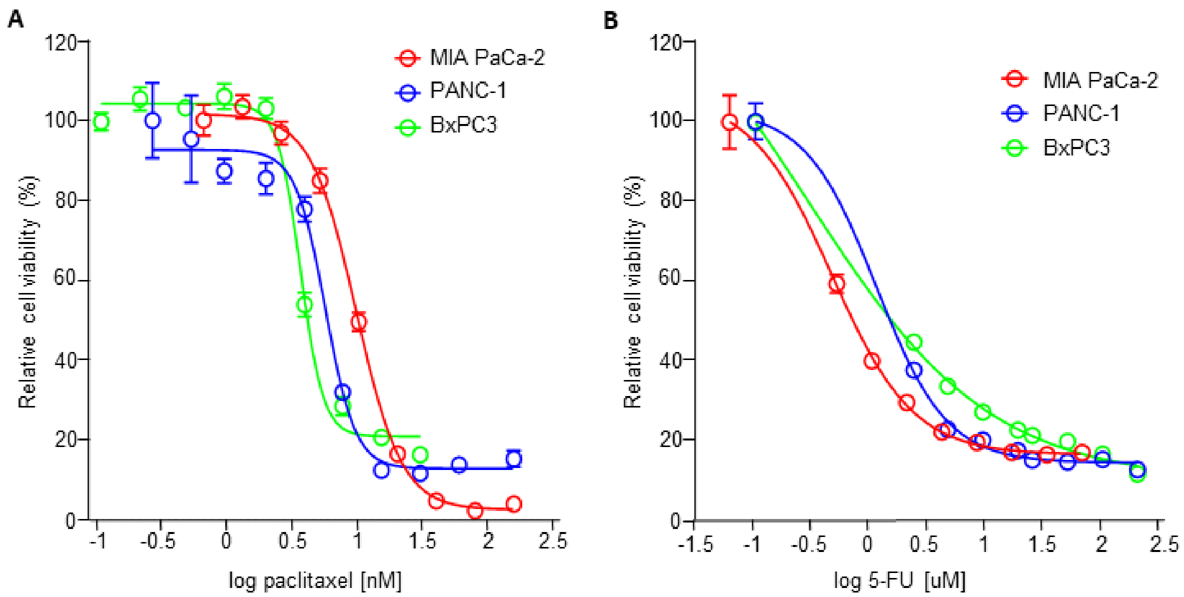

Supplemental Figure S3: Dose-response curves of paclitaxel and 5-FU treatment. Cell viability of Mia Paca-2, BxPc3 and PANC-1 cells at 96 hours of paclitaxel (A) and 5-FU (B) exposure. Shown is the mean±SEM of representative experiments performed in sixplo.
